# Using Machine Learning to Efficiently Vaccinate Homebound Patients Against COVID-19: A Real-time Immunization Campaign

**DOI:** 10.2196/37744

**Published:** 2022-07-12

**Authors:** Anish Kumar, Jennifer Ren, Katherine A Ornstein, Peter M Gliatto

**Affiliations:** 1 Icahn School of Medicine at Mount Sinai New York, NY United States; 2 Department of Geriatrics and Palliative Medicine Icahn School of Medicine at Mount Sinai New York, NY United States; 3 Institute for Care Innovations at Home Icahn School of Medicine at Mount Sinai New York, NY United States; 4 Department of Medicine Icahn School of Medicine at Mount Sinai New York, NY United States

**Keywords:** home care, covid, vaccination, COVID-19, machine learning, vaccine, geographic cluster, patient data, clustering algorithm, geospatial, digital surveillance, public health, logistic operation, electronic health record integration

## Introduction

In March 2021, the New York City Department of Health and Mental Hygiene (NYCDOHMH) received a supply of single-dose Janssen COVID-19 vaccines to vaccinate the city’s patients who are homebound, but they needed assistance to identify and reach this population [1,2]. Mount Sinai Visiting Doctors (MSVD) is a large home-based primary care program serving more than 1200 patients who are homebound throughout Manhattan. We partnered with NYCDOHMH to vaccinate patients in our program. The administrative team generally schedules routine home visits based on zip codes, using 12 unique catchment areas covering all of Manhattan. This existing zoning system was inadequate for vaccination purposes for several reasons, mainly because these zones were too large. Furthermore, the additional task of manually scheduling patients by zone would be overwhelming for administrative staff, especially given the temporal constraints of the Janssen vaccine (ie, doses expired in 6 hours). In response, we developed a system to geographically cluster patients to efficiently vaccinate our homebound patients against COVID-19.

## Methods

### Ethics Approval

The Icahn School of Medicine at Mount Sinai’s Program for the Protection of Human Subjects approved and granted a waiver of consent for this secondary data analysis study (STUDY- 21-00157) which was conducted in accordance with the Helsinki Declaration.

### Overview

We developed a software program that takes a cohort of unvaccinated patients in the MSVD Program as input and assigns each patient a cluster number. We used Python 3 (Python Software Foundation) [3] to process patient addresses, group them into clusters, and export these clusters into a standard database format and onto a map. Specifically, we used an open-source implementation of a modified unsupervised K-means clustering machine learning algorithm to group patients [4].

The first step was to convert patient households to spatial representations. Using the Google Maps Geocoding application programming interface (API), we obtained latitude/longitude coordinates of patient residences, which served as the input data to our algorithm.

These coordinates were then fed to the modified unsupervised K-means clustering algorithm. The standard K-means clustering algorithm clusters data points into *K* groups, while the modified algorithm allows one to impose constraints on cluster size, ensuring each group contains a number of data points within a specified range [4]. This was important for our process to avoid assigning an excessive number of patients to any one cluster; although many patients live close to each other, provider routes could not exceed 6 hours. Targeting approximately 5 patients per provider and bearing in mind that not all identified patients would desire and be available when the vaccine was offered by the scheduling team, we enforced a minimum of 12 and a maximum of 15 patients per cluster.

Next, we projected the data points onto a geospatial visualization for easy interpretability by the scheduling team and providers. Using the Google Maps API, we generated an HTML page that contained pins with distinct numbers and colors representing the cluster to which a patient belonged as well as the patient’s name.

**Figure 1:**
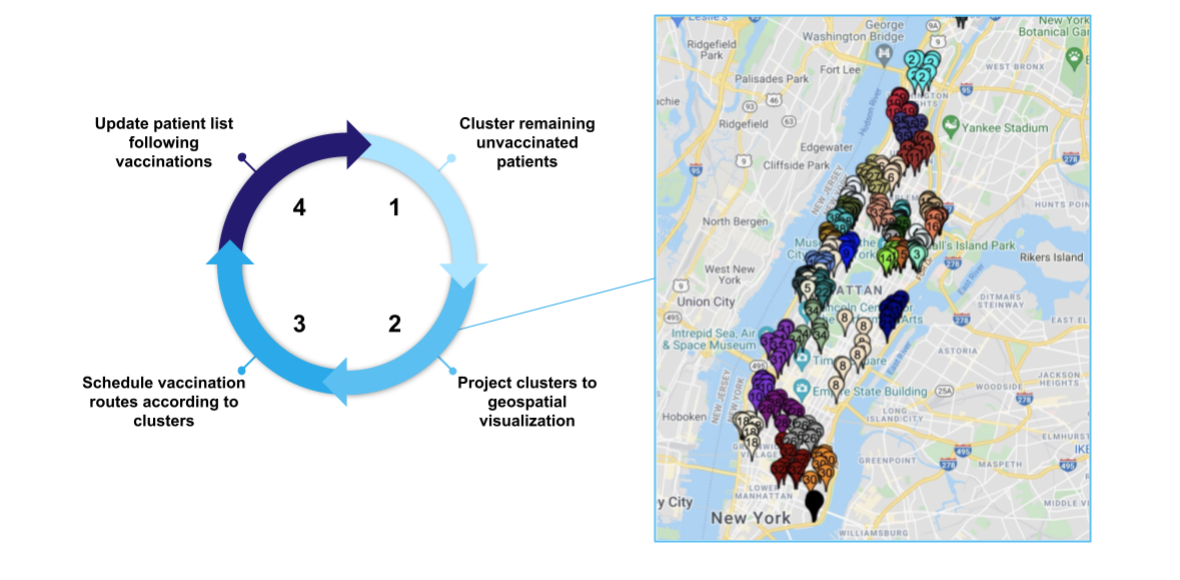
An example of a vaccination campaign, using mock patient data, for patients who are homebound in the Mount Sinai Visiting Doctors Program routing process. It was iterative week-to-week and included the following steps: (1) patients who were unvaccinated were identified and clustered into groups, (2) patient addresses were pinned on a map with identifying group labels, (3) patients were called for vaccine scheduling according to group number, and (4) the patient list was updated to reflect who remained unvaccinated.

Our process was iterative: every week after patients were scheduled and immunized, we updated our census to reflect the remaining number of patients who were unvaccinated. The team called patients to confirm appointments and updated the patient list. We then re-ran our clustering process, integrating any changes to cluster size preferences, for the next round of immunizations for patients who are homebound (Figure 1).

## Results

Table 1 summarizes the demographic information of the patients and the results of our campaign between March and April 2021, which involved about 100 vaccination clusters and routes over 6 weeks. On average, we vaccinated 5.6 patients per provider per day, averaging 22.1 total patients per day [5]. Each provider accomplished their assigned route within the time constraints of the Janssen vaccine, and no doses were wasted.

**Table 1:** Demographics and statistics from our COVID-19 homebound vaccination campaign of Manhattan-based patients in the Mount Sinai Visiting Doctors Program.

				Value
**Patient demographics**				
	**Patients who are homebound**			428
		Average age (years)		83.9
		Average Elixhauser comorbidity score [6]^a^		3.8
		**Sex, n (%)**		
			Female	323 (75.5)
			Male	105 (24.5)
		**Racial/ethnic identity, n (%)**		
			White	148 (34.6)
			Black or African American	63 (14.7)
			Asian	15 (3.5)
			Hispanic	108 (25.2)
			Other	86 (20.1)
			Unknown	8 (1.9)
	Patient’s family members and caregivers, n			92
**Vaccination campaign statistics**				
	Providers vaccinating per day (n), range			3-6
	Average number of patients vaccinated per day			22.1
	Average duration of provider time spent vaccinating (hours)			4.6
	Average duration of individual vaccination (including transit time, vaccine administration, and 15-minute postvaccination observation time; minutes)			52

^a^Elixhauser scores were available for 372 of the patients who are homebound.

## Discussion

The new tool optimized logistic operations for an acute public health intervention while minimizing wasted resources. The model was later used to quickly deploy booster immunizations during the surge of the Omicron subvariant from December 2021 to January 2022. Future research will consider the ability to create routes with ordered stops given a provider’s choice of transportation and more flexibility in the cluster size depending on the population density of a given region. This feature is especially important in densely populated cities, where providers are unlikely to travel by car. Additional future work can include electronic health record integration for more streamlined access and allowing scheduling teams to directly recluster patients.

Ultimately, the newly developed approach was instrumental in maximizing efficiency and minimizing vaccine waste, suggesting its potential for future use in home-based health care or other public health interventions. The success of this project further demonstrates the value of novel technological approaches in improving the efficiency of clinical operations.

## Abbreviations

API: application programming interface
MSVD: Mount Sinai Visiting Doctors
NYCDOHMH: New York City Department of Health and Mental Hygiene
